# Does information about toughness decrease fighting? Experimental evidence

**DOI:** 10.1371/journal.pone.0228285

**Published:** 2020-02-07

**Authors:** Aron Szekely, Diego Gambetta

**Affiliations:** 1 Collegio Carlo Alberto, Turin, Turin, Italy; 2 Institute of Cognitive Sciences and Technologies, CNR, Rome, Rome, Italy; 3 European University Institute, Florence, Florence, Italy; Universidad de Alicante, SPAIN

## Abstract

Will fights erupt when resources are scarce and the rules regulating their distribution are absent or ignored? We conjecture that the answer depends on whether credible information about individuals’ toughness is available. When people send credible signs and signals of their toughness disputes may be solved without violence. We use a laboratory experiment in which subjects create information about their toughness and decide whether to take others’ resources and resist in case others’ attempt to take theirs. Subjects perform a potentially painful but safe physical exercise to create information and to determine who wins and loses fights. This, realistically, ranks subjects according to their toughness and implicates toughness, a quality important in real conflict, in fighting. We find that, consistent with theory, information reduces fighting. This suggests that, in addition to the theories traditionally used to explain prisoner behavior, the availability of credible information about toughness influences prison conflict.

## 2. Introduction

Where resources are scarce and the rules regulating their distribution are either absent or unenforced—as is often the case in prison—does violence inevitably come to dominate interactions? The answer, we argue, depends on the availability of credible information about individuals’ toughness. Here we study the connections between information and violence experimentally.

Consider ‘toughness’ as the ability to fight and withstand fighting injuries before yielding to an opponent’s will. If individuals in conflict over resources have no information about each other’s relative toughness, we expect that disputes are likely to be settled with violence. If instead individuals have information about their opponents’ toughness we expect that disputes are more likely to be settled without violence [1]. When such information is available, weaker individuals do not challenge others and yield to the challenges of tougher opponents, while in its absence even weaker individuals may challenge others and risk a fight. The only case in which information does not help—as argued by the animal behavior literature—is when individuals are of a similar toughness, thus information cannot solve disputes and violence ensues.

By settling disputes through information rather than violence both weaker and stronger individuals gain: the former avoid the harm caused by violence and, we conjecture, are no more exploited than in the absence of information; the latter too gain for they do not incur the cost of meting out violence, and avoid the injuries that even weaker opponents may inflict.

Information on an individual’s toughness can be transmitted through *signals* or *signs*. Signals are actions intentionally taken to change the beliefs of others about one’s toughness. Like signals, signs carry information about people’s toughness, but unlike signals they are not produced and displayed with the intention of informing present opponents; signs include information about past actions, sometimes transmitted by the perceivable traces they leave on the body or the demeanor [2]. In our experiment, subjects are given the opportunity to build both signs and signals of toughness.

To reduce violence, information needs to be *credible*. As posited by signaling theory [3,4], a signal of toughness is credible if only truly tough signalers can afford it, while none or few weaker signalers can. In this way ‘dishonest’ signals are impossible or rare allowing receivers to infer their opponents’ toughness [3–5]. A sign of toughness too derives its credibility from cost discrimination—for instance, cauliflower ears are credible signs of having endured past physical conflict. However, signs are often noisy: a scar or a broken nose can indicate a boxer or a careless driver [2].

Signaling theory not only proposes these conditions but also predicts how different types of people should signal [3,4]. A core proposition of the theory is that people with a high level of a quality, toughness in our case, signal with a higher intensity than people with a lower level of the same quality. We test whether this holds in our experiment.

We also test an extension of the standard signaling model, called countersignaling theory [6]. This introduces two variations: instead of two types of people, it posits three–low, medium, and high quality—and assumes that some exogenous noisy information already gives information to receivers before they receive any signals. ‘Signs’ in our experiment embody this exogenous information. With these modifications, a different prediction emerges. Medium toughness people signal with the highest intensity while both the weakest and the toughest signal less. Medium types signal to avoid being mistaken as weak, weak types signal little because signaling is too costly for them, and the truly tough also signal little, they ‘countersignal’, to avoid appearing like over-eager mediums.

We use a laboratory experiment, tailored to studying prison fighting, to examine these questions. In addition to economic incentives that are standard in behavioral experiments, we implicate the quality of toughness in determining who wins and loses a fight using a ‘competitive wall sit’. This, realistically, means that both winners and losers incur pain from fighting and that the winner takes the resources of the loser. Although our focus is on prison conflict, we aim to contribute to the literature on conflict more broadly. By studying the extreme setting of prisons, we seek to understand conflict inter-personal conflict more generally.

Prisons offer an ideal setting in which to study our questions because resources are scarce, rules are absent or weak, prisoners encounter many unknown other prisoners, and the negative consequences to interactions can be extreme. As a result, prisoners have powerful incentives to engage in meticulous strategic reasoning [1,7,8]. They are distilled microcosms of conflict in the outside world. Indeed, our conjecture originates from a study on prisons [1].

### 2.1 Related literature

The fitness advantage of solving disputes through credible signaling of fighting ability is well-established in the literature on other species [9–12]. But the connection between information and fighting has hardly been investigated among humans and only once experimentally [13]—even among other species, experiments on information and fighting are rare [but see 14,15].

The other existing experiment, by Przepiorka and colleagues [13], uses a variant of the hawk-dove game to test how different information conditions shape the emergence of dominance hierarchies. They predict, using a game theoretic model, and experimentally find, that credible reputation of ‘fighting’ ability eventually reduces conflict and that dominance hierarchies naturally emerge when such reputation can be transmitted.

While their results are consistent with what we find, there are two important distinctions relative to our study. First, we test how signs and signals that are created outside of a conflict interaction shape fighting in one-shot situations. Conversely, Przepiorka et al. consider richer situations in which subjects repeatedly interact, and, how the information generated through repeated interaction shapes conflict later. Second, we model interactions in which one person is the ‘senders’, who emits the signs and signals, and the other is the ‘receiver’, who perceives the signs and signals and can respond, but does not himself send any information. Przepiorka et al., instead, model interactions in which both people are simultaneously senders and receivers observing each other’s information and responding to it. Thus, our experiments model different but complementary situations.

The other closest experiments come from Benard [16,17] who focuses on reputation systems. A core hypothesis, for which he finds consistent support, is that reputational systems, which support information transmission, increase aggression because it incentivizes subjects to build up reputations to deter and intimidate others. Although these experiments are unable to test how reputation systems affect outcomes because subjects only interact against simulated partners who play predetermined strategies, they do reveal the effect that reputation has on actions because participants believed they were facing real people.

Even non-experimental research on the relationship between information and fighting in humans is sparse. A review of the psychological literature, which is rife with studies on conflict, yields no directly relevant work. The sole human context, as far as we have been able to establish, in which this has been studied is prison. Evidence from a range of qualitative and quantitative sources lends support for the effect of information on prison violence and shows that variations in interpersonal fights are consistent with variations in information [1,8]. While promising, however, this evidence rests on observational data. In this paper we test whether some of these connections hold in an artificial but controlled experimental lab setting.

A few further works develop related ideas. In *Collision of Wills* [18], the late sociologist Roger Gould, holds that fights over status are more likely to occur in ‘symmetrical relations’, for instance, amongst brothers, friends, and peers, than between individuals who are part of hierarchical organizations in which rank is clear. ‘Symmetrical relations’ could be taken as an approximation of ‘similar fighting ability’, and hierarchical rank as one of the several forms in which resource distribution is normatively governed. Robinson [19], too, builds a model of social identity and conflict and predicts that, generally, inter-ethnic conflict is greater when the groups have more equal resource distributions. In the political science literature, Fearon [20] argues that a lack of information can drive war. Yet, his work is applied to state-level conflicts and not to inter-personal interactions.

More generally, there is a rich theoretical and empirical literature on conflict games (e.g. the chicken game [21] and the chain store game [22–25]) and economic contests [26–28]. Some of these theoretical models, such those on the chain store game [24,25], make predictions similar to ours: predatory pricing—economic aggression—occurs only when there is uncertainty about type of the players involved. However, these studies do not empirically test the causal effect that information has on aggression and conflict and do not implicate people’s real toughness in the interaction.

Concerning signaling, the theory was developed in economics [3,4] and biology [29,30], and is now used across the spectrum of social sciences [see 2]. While there are now many models of signaling [e.g. 31–33] and empirical applications [e.g. 34–36], experimental tests remain sparse. Nevertheless, the handful of experiments that do test signaling theory [37–40] find clear support for its predictions. In contrast, only two experiments test countersignaling theory [6,41] and find ambiguous results. In our experiment, we implement one treatment (the signal treatment) that supports only signaling and another treatment (the sign+signal treatment) that tries to capture the countersignaling model’s conditions. This allows us to assess whether countersignaling emerges in an environment that provides fertile ground for it.

Finally, deprivation and importation theory are the two theories traditionally used to understand prison fighting [e.g. 42–46]. The former posits that fighting is a function of the ‘pains of imprisonment’, while the latter argues that it is the culture acquired by individuals outside of prison that determines it [47–50]. In our experiment, we control for these theories’ posited factors as our subjects experience the same deprivations, and since they are randomly allocated to treatments and come from a pool of similar students, they should import broadly the same culture.

## 3. Methods and hypotheses

### 3.1 The prison entry game

**Fig 1:**
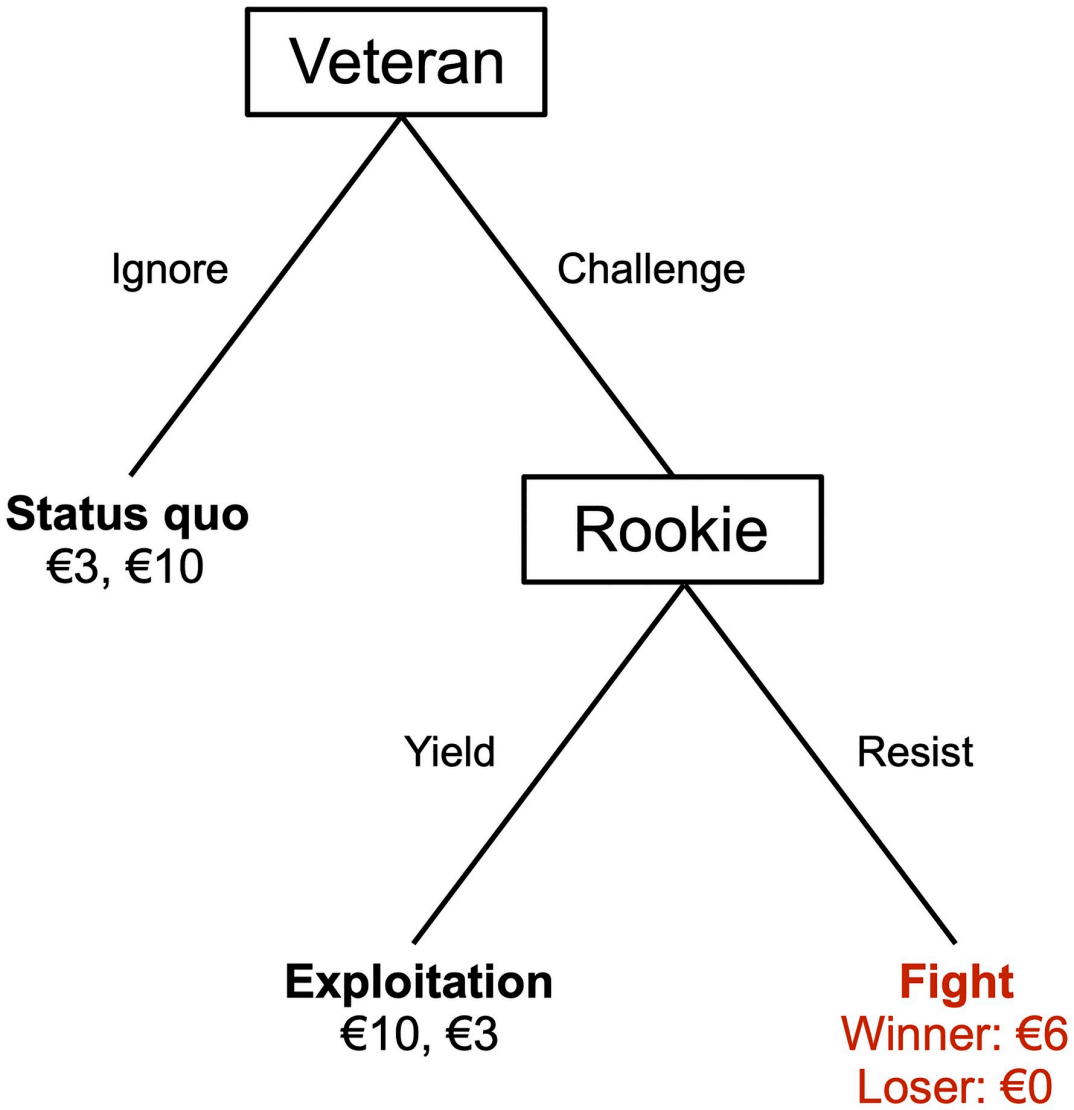
Our prison entry game.

To reproduce in the lab the essence of a typical prison situation in which fighting over resources may occur, we introduce two characters and make them play a game (the ‘prison entry game’) (Fig 1). Although in the experimental instructions we refer to these characters using abstract terminology, consider them as established prisoners, ‘veterans’ (V), and as newcomers to that prison, ‘rookies’ (R). We assign more resources to R (10) than to V (3) to induce through their payoffs the incentives that real prisoners typically have to covet the resources of newcomers. V decide whether to *ignore* or *challenge* R try take their resources. If V choose to ignore, this maintains the status quo, so V retain 3 and R keeps 10 (for whom this is the preferred outcome). If V choose to challenge R, R then decide whether to *yield* or to *resist*. If R yield, this leads to exploitation and R are left with 3. While this is the preferred outcome of V, who thereby reverse the resource allocation and obtain 10. If R *resist* a fight ensues (we describe in Section 3.2.1 how fights are settled). Losers get nothing, but winners too pay a cost, whether they are R or V and receive only 6. Overall, fights imply an efficiency loss relative to status quo or exploitation since the combined outcome from fighting (6+0) is less than the combined outcomes from status quo (3+10) and exploitation (10+3).

Our game has much in common with standard models of conflict (e.g. the chicken game [21] and the chain store game [22]). Indeed, it is a modified version of the entry game [e.g. 51]. Differently to the entry game, however, in which the newcomer to a market first decides to enter or not, V move first. This is because the more common situation is that in which established prisoners covet, and want to take, the resources of newcomers. Additionally, in contrast to other models of conflict, one of our outcomes, namely fighting, incorporates a physical exercise in which tougher individuals are likelier to win (we describe later in detail how fights are resolved in Section 3.2.1). This, realistically, implicates toughness in our conflict interaction.

### 3.2 Design and treatments

To test the role of credible information on aggression in the prison entry game, we use a three-step procedure [see also 38,41] and we randomly allocate subjects to one of three information treatments (described below) in a between-subjects design. Subjects first generate a sign of their toughness by doing a ‘wall sit’ exercise for gain (every second they stay in the position they earn €0.02). The wall sit is a non-harmful but pain generating exercise that assesses subjects’ toughness (described below in Section 3.2.1). Subjects do the wall sit here without knowing the details of the experimental stages that follow, and thus, they do not wall sit to create information but to earn rewards. This stage, which we call the *veiled sit*, allows us to measure subjects’ natural toughness without polluting it with strategic concerns. While we do not inform subjects that their performance in the veiled sit may affect their payoffs in the following stages, they are aware that further stages follow, and more importantly, we do not tell them that their performance will have no effect on their payoffs. Such a ‘reveal’ method is consistent with experimental best practice and is used in multiple other experiments [41,52,53].

Next, the details of the experiment are unveiled, and everyone learns:

(i) that their time in the veiled sit is used to assign them a grade: those wall sitting for the longest 1/3rd are allocated an A, those in the middle 1/3rd receive a B, and those in the bottom 1/3rd receive a C (converting wall sit prowess into grades representing tertiles ensures that participants have common priors about the distribution of types. Simply transmitting times in seconds would not allow this);
(ii) which grade they obtained in the veiled sit; and
(iii) how the prison entry game works.

Furthermore, we tell them that:

(iv) they will play the prison entry game a number of times, both as V and as R, with randomly matched partners whose identities are mutually anonymous;
(v) before playing the game they will redo the wall sit, this time unpaid but with full knowledge of the details of the experiment; in this way, subjects can create a signal of their toughness (*unveiled sit*);
(vi) they will receive another grade A, B or C, for their performance in the unveiled sit allocated relative to the veiled sit times of their session; subjects whose unveiled wall sit time falls in the range of times for which a given grade was assigned in the veiled wall sit, will get that grade (we decided to specify unveiled wall sit grades this way because this procedure realistically allows R to have any distribution of signaling levels. Splitting unveiled grades into tertiles would have unnecessarily and awkwardly restricted their signals into three equally distributed categories.);
(vii) if in the game challenging by V is met with resistance from R, there will be a ‘fight’ consisting of a third wall sit in which the fighters simultaneously wall sit and the winner will be the person who endures longer in the position; and
(viii) they will be paid randomly for one of their decisions as either V or R.

**Fig 2:**
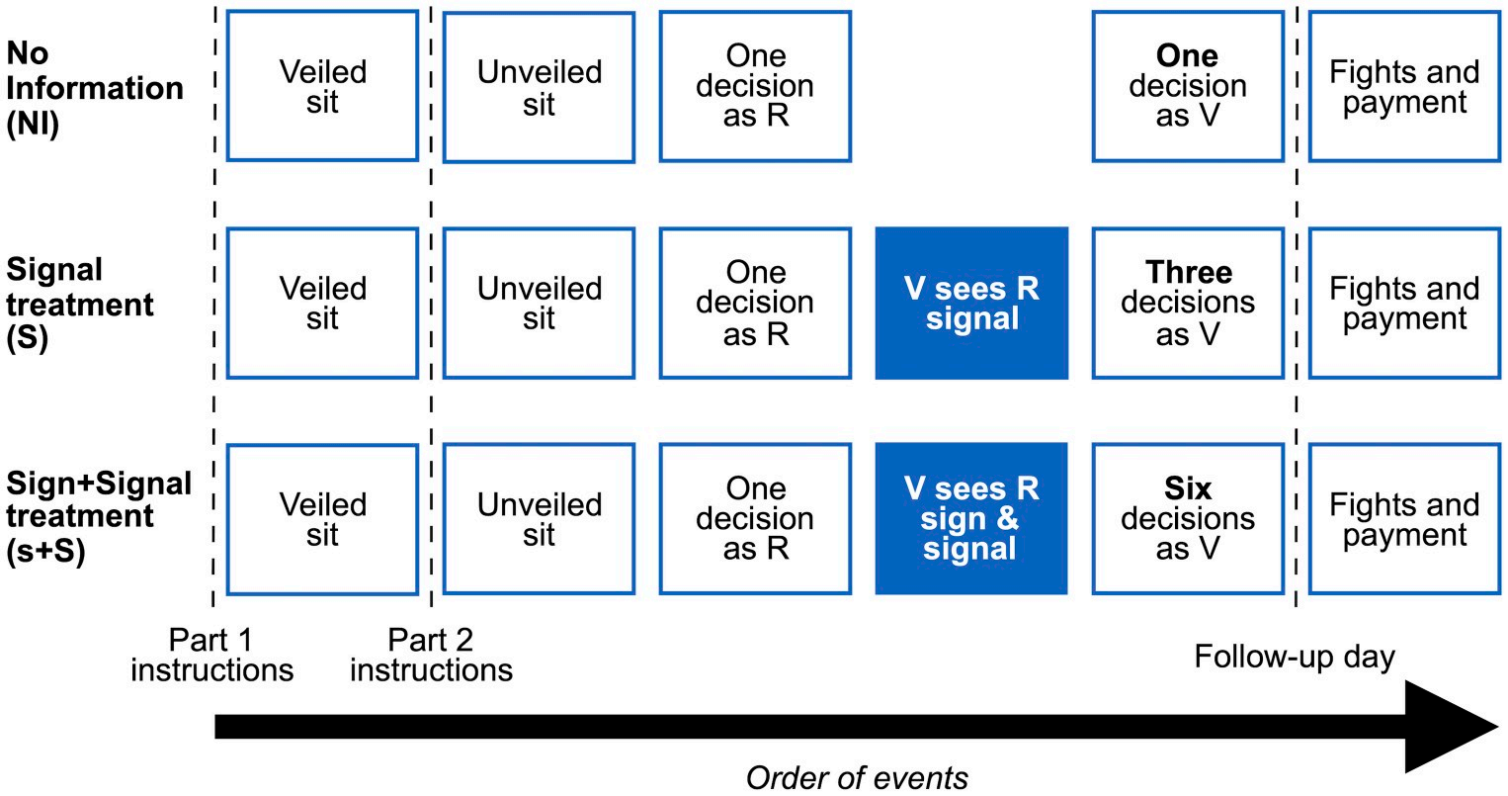
The design and treatments.

In the *no information treatment* (NI) this is all that subjects are told (Fig 2).

What additional instructions subjects receive in the information treatment depends on which of two variants they are in. In one variant subjects are told that when playing as a R, the grade they obtain in the unveiled sit will be revealed to the V they are partnered with before the V decides whether to challenge—this is the *signal only variant* (S) (Fig 2). This capture the common situation in which prisoners know nothing of each other’s previous displays of toughness, and the information they exchange is predicated on signaling actions that they take in prison while observing each other.

In the other variant they are told that in addition to the signal (their unveiled sit grade), their partnered V will also be informed of their sign, namely their veiled sit grade, before deciding how to act. And, furthermore, that the veiled sit grade, as is typical of signs, will have some ‘noise’ injected into its transmission that eliminates B grades: before being sent, B veiled grades are transformed into A with 0.5 probability and C with 0.5 probability. This is the *sign and signal variant* (s+S) (Fig 2). This capture the situation in which, in addition to the signal intentionally produced, some exogenous information is known about new prisoners. We do not have a sign only variant since in real life it is less likely that V make their decisions based only on exogenous information, while in *most* conceivable encounters R have an opportunity to signal to V before a challenge is levelled. Gladiators may or may not know about their opponents’ previous deeds, but on entering the arena to fight they can *always* take actions to signal their toughness to each other.

Only V ever receiver information about the signs and signals of R and not *vice versa* in our experiment. As established inmates, V already know the toughness of other prisoners they are incarcerated with. This allows V to focus on ‘reading’ the signs and signals of the newly incarcerated R. In contrast, as newcomers, R are faced with a multitude of unknown V all of whom have different signs and signals. This makes it difficult for R to interpret the signs and signals of any specific interlocutor and hence R lack intelligible information. In our idealized interaction, it is not that V possess few signs and signals—they may have an abundance of them—but that R entering a prison are overwhelmed making it difficult, if not impossible, for them decipher the symbols of any particular V.

While we use the labels veterans and rookies to elucidate our interaction, these role names should be considered as ‘stand-ins’ that are more precisely defined by the distribution of information between the players: one of whom can have information about the other while the other never does. Thus, our situation applies just as well to two established prisoners in conflict—one of whom has information about the other, say from a newspaper cutting, or because the other has recently moved institution, and to two rookies in conflict—one of whom knows something relevant about the other.

In the information treatments R have an incentive to signal and to do so honestly. This is ensured by a dual pressure. On the one hand, the payoff structure is such that tougher R always want to be perceived as tougher types for they strictly gain from deterring V than from fighting. On the other hand, the signal is so costly that only truly tough R can afford to send the highest grade. Also, V have incentives to accurately assess the toughness of R accurately. Misidentification of toughness—both too high and too low—implies either a missed opportunity or a waste of resources, energy, and pain in a fight.

We use the strategy method to elicit subjects’ choices [54,55]. This allows us to obtain sufficient observations on the decisions of V against all possible combinations of R signs and signals—even for those, such as two A grades, that may be rarely observed. The downside is that we do not directly observe the outcomes that occur between interactants; to overcome this we use simulations to repeatedly match subjects and predict what would happen (see Section 4.2).

R make a single choice to resist or yield, and V make a decision to challenge or ignore for each of the possible sign-signal combinations, up to six depending on the treatment. Specifically, V make one decision in the no information treatment, for there are no signs and signals to observe, three decisions in the signal variant—one for an A signal, one for a B signal, and one for a C signal—and six decisions in the sign and signal variant: one for each of the three signals combined with either a C sign or an A sign. The order in which the combinations are shown to V is randomized across sessions.

To avoid tiring subjects, we split each experimental session in two parts, the experiment proper and the follow-up, held a few days later (Fig 2). In the former, subjects carry out the veiled and unveiled sits, and make all of their decisions. In the latter subjects are told their outcomes and if this was a fight, they participate in the competitive wall sit to decide the winner and then receive their payment. The others are paid right away. In order to avoid personalizing the fight and breaching the anonymity of the experiment, those who end up fighting do the wall sit while wearing sleeping masks and ear protectors just as they did in the main session.

#### 3.2.1 Measuring toughness and settling fights: The wall sit

We use a non-harmful pain generating ‘wall sit’ to assess toughness. In this exercise, subjects bend their knees and lean against a wall such that their shins are parallel to the wall and their thighs are parallel to the floor. Wall sitting has been used a few times in experiments to measure altruism, the trade-off between pain and gain, and to test whether enduring pain promotes in-group cooperation and is related to the number of friends [56–61]. It has never been used to measure toughness.

To represent fights in the experiment, we use a competitive wall sit. Here, subjects simultaneously wall sit and the winner, the person who endures longer in the position, takes the loser’s money. In contrast to prior experiments on conflict, which typically use self-reported experience of fighting [62–64] or fighting as a potential loss of money [13,16,65–67], our competitive wall sit captures key elements of real fights: it can be painful and requires both physical resilience and toughness.

To conduct the veiled and unveiled sit, subjects were taken in random groups of 4–8 in a large lecture room and asked to wall sit simultaneously. To prevent them from perceiving each other’s wall sit times they wore sleeping masks and ear protectors. Each wall sit session gave subjects the opportunity to stay in position for a maximum of six minutes, and this was public knowledge; only after this time span, regardless of whether anyone was still wall sitting or simply resting sitting on the floor, the session was declared concluded. This prevented the subjects in the same group from inferring what the longest wall sit time was based on how long they remained in the room. Subjects returned to the experimental lab following their wall sits.

Given the physical nature of the experiment, we undertook extensive precautions to mitigate possible risks. These included highlighting the physical nature of the experiment in recruitment material and requesting that people with back or knee problems not register; re-iterating multiple times during the experiment that subjects should not participate if they have back or knee problems and that they are free to leave and would receive their show-up fee; keeping a careful watch on subjects while they wall sat; explaining the purpose of sleeping masks and ear protectors, and ensuring that there were always pillows underneath wall sitting subjects.

To validate wall sitting as a measure of toughness and as a model for fighting, we record a set of variables that may express toughness and pain resilience in other ways: we ask subjects to rate their fitness, frequency of exercise, and ability to withstand pain; and to answer the Buss-Perry Aggression Questionnaire (BPAQ), a widely used tool in psychology to measure aggression [68]. We also measure the ratio between the length of 2^nd^ digit and that of the 4^th^ digit (2D:4D) on the right hand of subjects, which has been found to correlate with a variety of aggressive behaviors in men [69,70].

Using veiled wall sit times, since all subjects at this stage have the same incentives, we find in a multivariate linear regression that wall sit time, that is how long subjects maintain the position, is related to pain resistance (b = 16.55, s.e. = 5.15, p = 0.002), fitness (b = 12.90, s.e. = 6.03, p = 0.034), 2D:4D (b = -221.19, s.e. = 104.20, p = 0.035), and body mass index (b = -2.86, s.e. = 1.55, p = 0.068) but not related to the BPAQ, frequency of exercise, and age (S1 Appendix). There are no issues of multicollinearity (all VIFs are below 2). Thus, wall sitting captures toughness and requires attributes that are present in real fighting. Greed and need, which we do not record, could add noise to the measure since those with a greater desire for money may wall sit for longer. However, these dispositions should be randomly distributed across toughness grades and treatments, and hence should not affect our results.

### 3.3 Hypotheses

In our imagined prison, inmates, new and old alike, are divided into three grades of relative toughness: tough (A), medium tough (B) and weak (C), each corresponding to a third of the individuals. We refer to V and R of varying grades, and who know they possess these grades, with V_X_ and R_X_ where X is either A, B, or C.

Consider now *information* concerning toughness. We assume that both V and R always know their own toughness grades. Then we have two conditions: in the first condition V do not know the grade of R, and R know that this is the case (‘no information’ condition). In the second condition, V know the grade of R, and R know that this is the case (‘information’ condition). By contrast, we assume that as newcomers to a prison in neither condition R know anything about the toughness of the specific V they face, although they hold uniform prior distributions about the toughness of the V in the population and this is the case in the experiment.

V come to know something about the toughness of R through *signs* of grade—indirect information on actions R took that reveal something of their toughness, e.g. the crime R is jailed for, previous prison records, previously acquired fighting reputation that are often advertised through prison grapevine or other media, and *signals*—actions that R take in order to show V their toughness.

We make two hypotheses concerning agents’ decisions in the interaction. When V have credible information on the toughness of R, *V challenge less* (H1). This, in terms of outcomes, implies that the status quo increases to the advantage of R. Next, when V have information on the toughness of R, *R resist the same or less* (H2). If the changes in decisions that we hypothesize in H1 and in H2 both occur, we predict that outcomes should vary thus: exploitation may increase but could also remain the same and not get any worse; most importantly, fights decrease, with the result of generating a more efficient overall outcome.

Here we describe how subjects might reason in making their decisions in our experiment, leading to our hypotheses, and for simplicity we focus on the two extreme toughness types, A and C. The formal models whence our hypotheses derive, is in S2 Appendix, and the predicted actions of V and R are shown in Table 1 while the hypotheses are specified precisely in Table 2. We model the two conditions using games of asymmetric information and solve for pure strategy perfect Bayesian equilibria to derive the predictions. Remember, we refer to V and R with varying grades using V_X_ and R_X_ where X is either A (the toughest), B (intermediate), or C (the weakest).

**Table 1:** Predicted actions of V and R according to their types and setting.

V and R types	Predicted actions	
	No Information	Information
V_A_	Challenge	Challenge or Ignore R_A_, Challenge R_B_, Challenge R_C_
V_B_	Challenge	Ignore R_A_, Challenge R_B_, Challenge R_C_
V_C_	Challenge	Ignore R_A_, Challenge R_B_, Challenge R_C_
R_A_	Resist	Resist
R_B_	Yield or Resist	Yield
R_C_	Yield	Yield

V_A_ are indifferent between challenging or ignoring R_A_ in the information setting and R_B_ are indifferent between yielding or resisting in the no information setting.

**Table 2:** Our hypotheses about V and R actions and the resulting outcomes.

		No Information	Information
**Actions**	Challenging decreases with information (H1)	100%	67% to 78%
	Resisting stays the same or decreases with information (H2)	33% to 67%	33%
**Outcomes**	Status quo	0%	22% to 33%
	Exploitation	33% to 67%	67%
	Fight	33% to 67%	0% to 11%

Numbers indicate the percentage of people predicted to choose an action or to end up in an outcome. Outcomes in the No Information condition can be directly calculated from the chosen actions (e.g. 1*0.33 and 1*0.67). The situation is more complex in the Information condition since challenging is conditioned on the grades of R and V.

Start with V_A_ (Table 1). Without information on the grades of R, V_A_ expect to win all fights except a fraction of those against R_A_. This encourages V_A_ to challenge all R since the gain from ignoring are low and benefits of exploiting are sufficiently high to risk fights—which in any case V_A_ wins in all cases except when facing R_A_. With information on R, V_A_ will challenge R_B_ and R_C_, but when facing R_A_, who are as tough as they are and thus expected to resist, V_A_ will be ‘indifferent’ between ignoring and challenging. With information, therefore, V_A_ make at most similar or fewer challenges.

Consider now V_C_ (Table 1). With no information on R, V_C_ expect to lose most fights (all those against R_A_ and R_B_ and some of those against R_C_). But strategic V_C_ anticipate that all R_C_ and some R_B,_ afraid of encountering and resisting against V_A,_ will yield and thus challenge R hiding behind the fear created by V_A_. When information about the toughness of R is present, by contrast, V_C_ does not challenge R_A_, for they know they would lose in a fight. (V_B_ may follow the reasoning of either V_A_ or V_C_ in the no information treatment and the reasoning of V_C_ in the information conditions). Our first hypothesis therefore is that *with information there are fewer challenges* (Table 2). In terms of outcomes, this implies that with information *there is an increase in status quo* to the advantage of R.

Consider now the perspective of R_A_ (Table 1). Remember that R are never told the grade of V, but R know whether or not V are told the grade of R. Hence R know whether the decisions of V rely on that information. When V have no information on R, R_A_ choose to resist all challenges as the probability that they win in any fight is so high (they expect to always win against V_B_ and V_C_, and to win some of the time against V_A_). When V has information on R grade, R_A_ may anticipate that most V who challenge are of A grade but also a few weaker V who bluff. Since R_A_ win against V_A_ some of the time and always against V_B_ and V_C_, then their reasoning could be that on average it is to their advantage to resist; the only time that R_A_ might hypothetically consider yielding is if only V_A_ challenged and all others ignored, which never happens in equilibrium.

Finally, consider the perspective of R_C_ (Table 1). When V has no information on the grade of R, R_C_ should choose to yield every time, as they expect to lose most fights. When V has information on R grade, nothing should change and R_C_ should also yield all the time expecting to lose most fight. Our second hypothesis is in two parts: *With information resisting stays the same*. However, if we consider the behavior of R_B_ *resisting may decrease* (Table 2). This is because in the absence of information R_B_ are indifferent between yielding or resisting, and so some proportion of them resist, while, when there is information R_B_ always yield.

In terms of outcomes (Table 2), we predict first as we said already that, when information is introduced, the status quo increases as a result of fewer challenges; next, that exploitation does not decrease, but it remains the same or increases. This is because when V has information both R_B_ and R_C_ yield and so the majority of interactions end in exploitation, but whether this level of exploitation is equal or greater than what happens without information depends on what R_B_ does: while it is clear that R_A_ resist and R_C_ yield, R_B_ may yield or resist. If R_B_ yield then most of the interactions end in exploitation, therefore the level of *exploitation is the same* in the two conditions since again both R_B_ and R_C_ are exploited. If R_B_ resist, by contrast only R_C_ are exploited. In this case exploitation is lower without information and *increases with information*.

For the third outcome, the above reasoning implies, most importantly, that *fights decrease with information*. The combination of fewer challenges and no change or decrease in resisting entails a reduction in fighting.

Our final hypotheses concern signaling and countersignaling theory. If signaling theory holds true in our information treatments then we expect that *tougher R signal more intensely than weaker R (H3)*. Instead, if countersignaling theory is the case, we predict that in our treatment in which R send both signs and signals, *tougher R signal less intensely than intermediate R (H4)*.

### 3.4 Participants

Our experiment was conducted at the Bologna Laboratory for Experiments in Social Sciences (University of Bologna, Italy) using their subject pool composed mostly of undergraduates at the University of Bologna. 198 subjects participated in our experiment across 12 sessions (between 16–18 in each). We recruited males between the ages of 18–40 as our subjects to reduce physiological differences in strength and fitness—those within this group are, on average, more similar to each other in isometric muscular strength and aerobic capacity [71,72].

**Fig 3:**
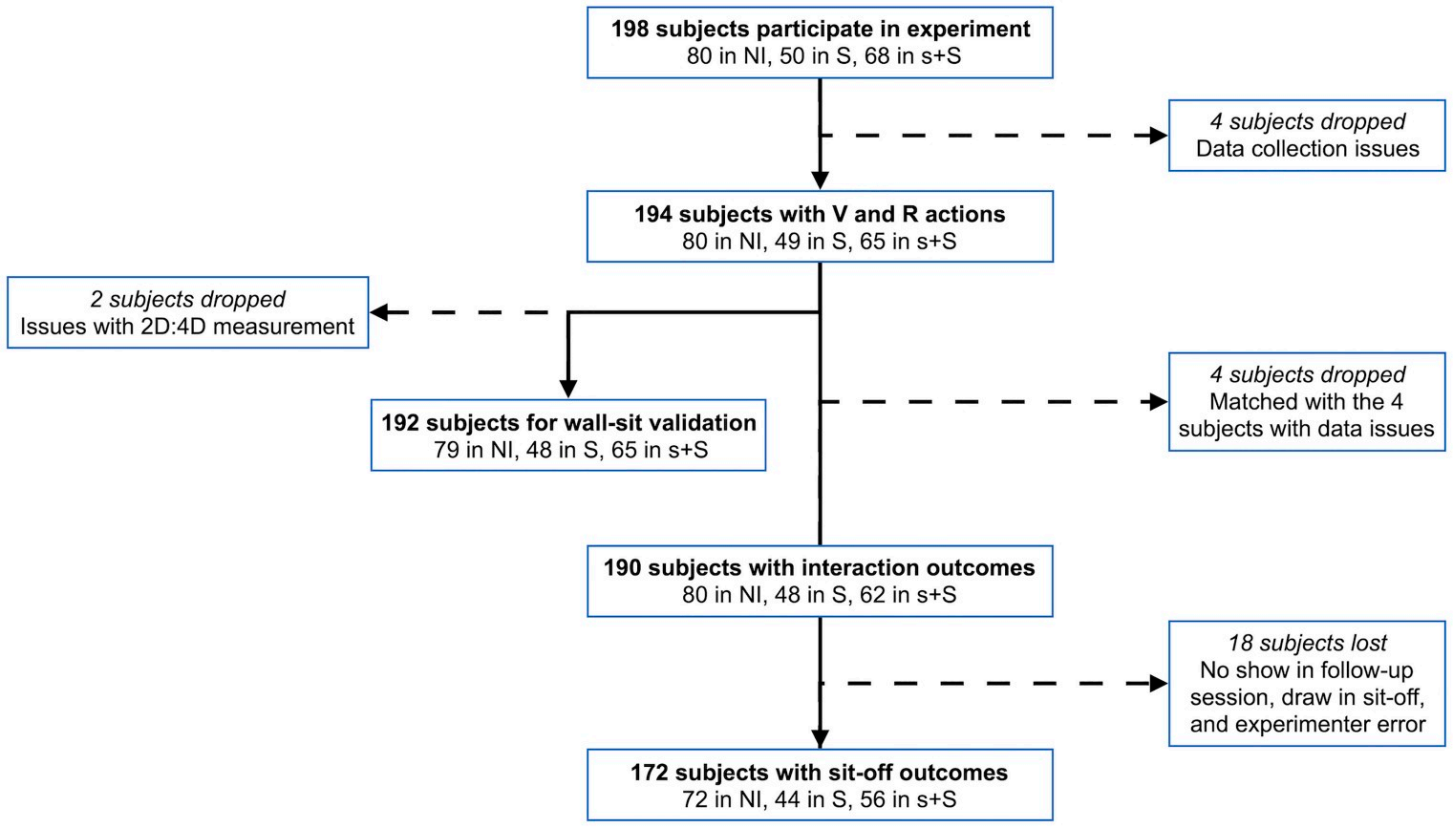
Sample breakdown.

Although 198 subjects participated in the experiment, we are able to use only 194 subjects’ sit times and actions as V and R (mean_age_ = 24.14, s.d. = 3.64). There are 80 subjects in NI, 49 in S, and 65 in s+S. For the actions of R, we have 80 observations in NI, 49 in S, and 65 in s+S, while, since the actions of V are elicited using the strategy method (V make a single decision in NI, three decisions in S, and 6 decisions in s+S), we have 80 (80x1) actions in NI, 147 (49x3) actions in S and, 390 (65x6) actions in s+S. We use all 194 subjects’ actions in our simulations to calculate the outcomes that occur. We also have data on 172 subjects for whom we know the outcome of any fights that occurred (See Fig 3).

The experiment was programmed with z-Tree [73] and analyzed using Stata v13.1 IC. The full outputs for models reported in-text are shown in S1 Appendix. The full experimental instructions can be found in S3 Appendix. Our data and code for the analysis and simulations are available online at https://osf.io/yuj7v/.

A pilot of our experiment was first evaluated and approved by the review board at the Centre for Experimental Social Science (University of Oxford, UK) in 2013. The final version of our experiment was again evaluated and received ethical approval from the Centre for Experimental Social Sciences review board in 2014. We conducted our experiment at the Bologna Laboratory for Experiments in Social Sciences (University of Bologna, Italy) which accepted the ethical review from the Centre for Experimental Social Science. Written consent was obtained from all participants.

## 4. Results

Before presenting the tests of our hypotheses, we check that (*i*) subjects understood the experiment and that (*ii*) wall sitting grade is a credible source of information about the toughness of R. With a few exceptions that are explicitly stated, we use logistic regressions to conduct our analyses. When analyzing the actions of V we use cluster robust standard errors at the subject level to account for the multiple observations per subject.

Multiple findings indicate that subjects understand the experiment. First, subjects answer 93% (1082/1164) of the control questions correctly. There are no between treatment differences: subjects answer 92.7%, 93.2%, and 93.1% of the control questions correctly in the NI, S, and s+S treatments respectively (none of these differences are significant). Second, they respond as expected to their own and others’ grades demonstrating that they properly understand the information value of grades in playing the game. Both V and R who possess higher grades, whether veiled or unveiled, are more likely to challenge (for V: veiled OR = 2.88, z = 6.64, p<0.001; unveiled OR = 3.47, z = 5.66, p<0.001; S1 Appendix) and to resist (for R veiled OR = 3.11, z = 5.54, p<0.001; unveiled OR = 4.48, z = 5.26, p<0.001; S1 Appendix). Third, V respond to both signs (OR = 1.58, z = 5.82, p<0.001; S1 Appendix) and signals (OR = 2.40, z = 6.78, p<0.001; S1 Appendix) of toughness by ignoring R with higher grades more; we consider only V in the joint sign and signal treatment (n = 65; 390 actions), for only they observe both sources of information. And, consistently with a broad signaling theory prediction, they respond more to signals of toughness than to signs of toughness since the latter are noisier, both because we add noise to signs and because higher signals are harder to produce (subjects are more tired during their unveiled sit) implying that only the truly tough are able to send B or A signals (OR = 1.52, z = 3.37, p = 0.001).

We also find that wall sitting resilience is predictive of the outcome of the wall sit ‘fights’ that may follow. The combatant with the longer veiled wall sit wins in 72% of cases (18/25 fights, Fisher’s exact test, p = 0.004), while subjects with the longer unveiled wall sit win every single fight (12/12 fights, Fisher’s exact test, p<0.001). This shows that wall sit grades are credible sources of information. With these conditions satisfied, we turn to our hypotheses, which predict what happens when V have credible information on the toughness of R.

### 4.1 Actions

#### H1: V challenge less with information

**Fig 4:**
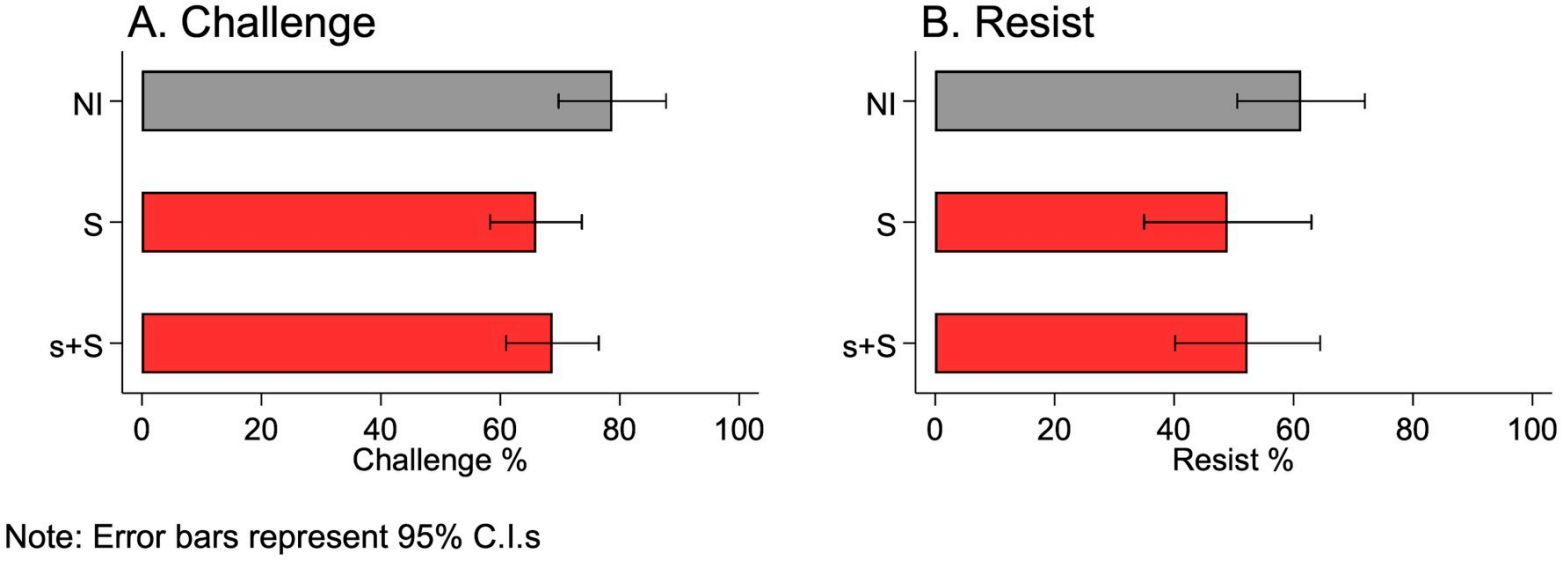
When there is information, (A) V challenge less and (B) R resist the same.

V challenge less in the treatments with information than in NI: 79% (63/80) of V challenge in NI while 66% (97/147) challenge in S and 69% (268/390) challenge in s+S. The difference between NI and S is significant (OR = 0.52, s.e. = 0.17, p = 0.046), while the difference between NI and s+S approaches the 10% significance level (OR = 0.59, s.e. = 0.20, p = 0.113) (Fig 4A; S1 Appendix).

#### H2: R resist the same or less with information

Resisting by R decreases when there is information (NI: 61% (49/80); S: 49% (24/49); s+S: 52% (34/65)), but these decreases are not significant (NI vs. S: OR = 0.61, s.e. = 0.22, p = 0.174; NI vs. s+S: OR = 0.69, s.e. = 0.23, p = 0.280) (Fig 4B; S1 Appendix).

### 4.2 Outcomes

Given the structure of the prison entry game, ignoring by V logically implies that status quo is the outcome, and, that fighting and exploitation are avoided. Assuming that the signs and signals of R in each treatment are equally distributed, a decrease in challenging thus implies that on average, there will be an increase in status quo and reduction in fighting and exploitation. What happens in our sessions, however, depends on the distribution of grades achieved by our subjects; while this is by design uniform for veiled grades, there is an overrepresentation of C and B in the unveiled grades of R. To determine the outcomes in our experiment, we randomly paired V and R once at the end of each experimental session and their decisions, which elicited using the strategy method, were implemented. We do not directly analyze these outcomes as it represents only what happens in one of many possible random pairings of subjects. Instead, to account for this matching variation, we run a simulation (using Stata v13.1 IC) in which we randomly and repeatedly pair subjects within each session and record the outcomes that occur. In two sessions there are an odd number of subjects; to deal with this, we randomly select one subject in those sessions to ‘sit out’ for one round.

Specifically, we run three simulations. Each simulation is used to explore one treatment: NI, S, and s+S. Every simulation is repeated 10,000 times. In each simulation round:

1. Every subject is partnered with a randomly allocated other subject within the same session.
2. One of each pair is randomly allocated to be V and the other to be R.
3. The outcomes are calculated based on V’s and R’s decisions, accounting, in S and s+S, for the grade of R and the grade observed by V.

#### Status quo does not change

**Fig 5:**
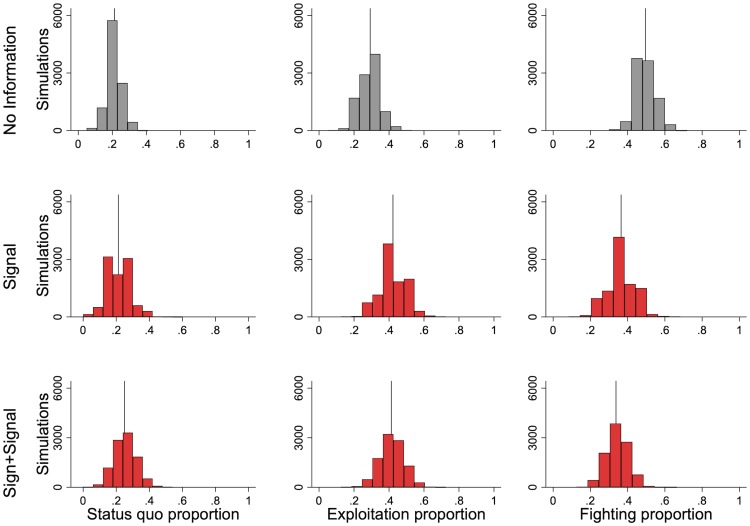
Information decreases fighting, does not affect status quo, and slightly increases exploitation in simulated outcomes.

Contrary to our prediction, the differences in challenging do not translate into differences in status quo (Fig 5). In NI 21.3% (s.d. = 4.5%) of groups end up in status quo while 21.3% (s.d. = 7.5%) and 25.0% (s.d. = 7.1%) do the same in S and s+S respectively.

Although we did not anticipate this lack of change, it is due to the large proportion of B and C grades that R obtain in the unveiled sit. In NI, since V cannot condition their behavior, the proportion V challenging directly determines the proportion of groups in status quo. In the information treatments, since V can and do condition their behavior, the distribution of R toughness matters for determining the outcomes. In these treatments, V challenge weak-looking R most of the time. Since most R obtain low unveiled grades—88% obtain a C or B (170/194)—lower-grade R are over-represented in the calculation of outcomes. This means that the R that V actually face for determining outcomes are challenged at a high rate and so status quo is lower than what would be expected by looking at average challenging alone.

#### Exploitation increases

Exploitation increases in the information treatments: it is 29.2% (s.d. = 6.1%) in NI, 42.3% (s.d. = 8.0%) in S, and 41.3% (s.d. = 7.3%) in s+S (Fig 5).

#### Fighting decreases

Fewer subjects fight when there is information: 50.0% (s.d. = 5.2%) of groups fight in the NI treatment while 36.5% (s.d. = 7.6%) and 33.7% (s.d. = 6.2%) fight, respectively, in the S and the s+S treatments (Fig 5).

Our findings so far are summarized below (Table 3).

**Table 3:** Summary of actions and simulated outcomes in the interaction.

		No Information	Signal	Sign+Signal
**Actions**	Challenge	79% [100%]	66%^p = 0.046^ [67% to 78%]	69%^p = 0.113^ [67% to 78%]
	Resist	61% [33% to 67%]	49%^p = 0.174^ [33%]	52%^p = 0.280^ [33%]
**Outcomes**	Status quo	21% [0%]	21% [22% to 33%]	25% [22% to 33%]
	Exploitation	29% [33% to 67%]	42% [67%]	41% [67%]
	Fight	50% [33% to 67%]	37% [0% to 11%]	34% [0% to 11%]

Square brackets show predicted percentages. Unconditional actions indicated. Superscripts show the p-values that arise from comparisons between the No Information treatment and the information treatments. There are no significant differences between the Signal and the Sign+Signal treatment.

#### 4.2.1 Fighting between equal and unequal grades

There is a possible exception to our finding that information reduces fighting. As the animal behavior literature argues, when individuals are of a similar toughness information cannot establish who will win and so fighting is likelier to emerge [74]. Similarly, Roger Gould [18] posited that violence is most likely to occur between people who are comparable in social rank, for instance among employees rather than between employees and bosses.

In our setting, both ideas imply that *fighting should decrease less when V and R are of the same grade*. When signs and signals cannot solve a deadlock, force is the only way to determine who gets the resources. While this conjecture is only partially drawn from the model, it is intuitive and well-established in the animal kingdom so we check for it here.

To establish whether subjects are of an equal grade or not, we use

- in the S treatment, the unveiled grades of V and R because unveiled grades are all that V observe about R; and
- in the s+S treatment, both their veiled and unveiled grades, for V observe both the sign and signal of R.

There is a complication in the procedure for the s+S treatment: R who obtain a B veiled grade cannot send this as a B sign since noise is injected into its transmission thereby turning a B veiled grade into an A or a C sign. When R obtain a B veiled grade, we calculate the sign that R actually sent V, then compare the sign of R to the veiled grade of V, and use this as our basis for establishing equality. For instance, if an R obtains a B veiled grade and once noise is introduced sends this as an A sign to V, then only if V has an A unveiled grade do we classify this as a match.

We assign no equal or unequal grades matches in the NI treatment because V know nothing about the grades of R, so the correct term of comparison is the overall proportion fighting in NI.

We use the same simulations as for calculating outcomes to determine the proportion of equal and unequal toughness groups fighting in each treatment. We find little support for different treatment effects among equal and unequal subjects. Remember that the simulated term of comparison in NI is 50.0% of subjects who fight (s.d. = 5.2%). Among equal grade V and R, there is a 12.9% drop in fighting in S (37.1%, s.d. = 12.0%) and 14.5% drop in s+S (36.5%, s.d. = 16.7%) relative to fighting in NI. Among V and R of dissimilar grades, the greater reduction is confirmed but the difference is small: fighting drops by 15.3% in S and by 17.1% in s+S (to 35.7%, s.d. = 12.6%, and to 32.9%, s.d. = 8.0%, respectively).

### 4.3 Signaling and countersignaling

Finally, we consider whether our data support the predictions of signaling or countersignaling theory. Consistent with signaling theory and H3, we find that veiled sit-times (our measure of toughness) and unveiled sit-times (R signal) are strongly positively correlated in the S and s+S treatments (S: r = 0.48, p<0.001; s+S: r = 0.63, p<0.001). R who obtain higher veiled grades in both the S treatment and the s+S treatment also wall sit for longer when unveiled (S1 Appendix). Additionally, we find no evidence in the s+S treatment that the toughest R signal less than intermediate toughness R (S1 Appendix). A grade R signal more than B grade R (b = 29.17, s.e. = 17.17, p = 0.094). Thus, H4, the countersignaling prediction is not supported.

Unexpectedly, we also find a positive association between veiled and unveiled sit-times in the NI treatment (NI: r = 0.57, p<0.001). This is unexpected since R have no incentive in this treatment to wall sit when unveiled; both because they earn nothing and V will not be informed of their wall sit times. Still, they resist in the position. We do not know why they do so. Maybe they are motivated by the competitive aspect of the wall sit or they want test themselves further to make better informed decisions in the game. Although we took steps to avoid a demand effect at this stage, by re-iterating that they need not wall sit and would be paid their show-up fee if they leave, it is also possible that they felt that they ought to wall sit. While it is not clear exactly why V in the NI treatment decide to sit when unveiled, them doing so does not affect our predictions, as, what matters is that in the other treatments there is a link between the wall sit grades and behaviors.

## 5. Discussion

In this paper we investigate experimentally the links between interpersonal conflict and information about individuals’ relative toughness. Our prison entry game captures the essence of a typical prison interaction in which fighting may occur, and, unlike prior experiments on conflict, which model fights with only monetary payoffs or rely on self-reported experience of fighting, we represent fighting using a competitive wall sit.

We find that how long subjects maintain the wall sit position is correlated with four other indicators of toughness which we also gathered: self-reported fitness and pain resistance, body mass index, and the ‘testosterone’ ratio between the length of 2^nd^ digit and that of the 4^th^ digit. These correlations validate our assumption that wall sitting captures attributes of toughness that are present in real fighting.

As for information, subjects create both signs and strategic signals of their toughness by performing a wall sit. This allows subjects to generate credible signs and signals since this exercise is a hard-to-fake indicator of toughness. Indeed, we find that in the case of a fight, the player with the longer veiled wall sit time wins in 72% of cases, while the player with the longer unveiled wall sit time wins every single fight. This shows that wall sit times report highly reliable information, and furthermore that signals are more accurate than signs.

In contrast to most signaling experiments [but see 38,41,75], subjects in our setting reveal their type; we do not exogenously allocate their toughness. This may lend itself to more natural in-game behavior and higher external validity because people who really are tougher play in the ‘tough guy’ role and because fighting in our experiment, like in reality, incurs some pain.

We add to the experimental literature testing signaling theory with our unusual wall sit method of toughness ranking and signal creation. Signaling theory’s prediction—that higher quality senders signal more than lower quality senders, is borne out in our data. Conversely, the prediction of the more nuanced and complex countersignaling theory that intermediate quality senders signal most, does not find support. Despite the setup of our experiment, which should be the ideal environment for the emergence of countersignaling, we do not find evidence of it. Perhaps the experimental lab is not the right environment in which to detect this behavior and subtle real-world qualitative investigations would be more able to identify countersignaling.

In terms of players’ actions, we find, as predicted, that V challenge less frequently in the treatment with information than in the control in which subjects play without knowing R’s grade. We also find that with information resisting by R decreases, though the difference is not significant. As for outcomes, we show that status quo remains the same, and that exploitation increases in the information treatments.

Above all, we find that fewer subjects end up in a fight when informed. Interestingly, we also find some evidence that the decrease is driven more by the interactions of players with unequal grades than with equal grades—the latter fighting frequency diminishes a little less than the former when information is given.

Overall, the increase in the frequency of the status quo implies that R are better off when information about their toughness is transmitted to V, who then are more careful to challenge. The small increase in exploitation indicates that R too are more careful to resist when challenged, and that thus V benefit from information on R’s toughness, at least they do not lose.

The conclusion is thus that if credible information about individuals’ toughness circulates well in situations in which fighting is likely to erupt, fighting should decrease and, to a varying extent, everyone should be better off, whether they would be winners or losers in fights. Even would-be winners gain by not fighting because fighting implies the risk of injury.

Our results also suggest that some amount of credible information is sufficient to reduce challenging and conflict. This is clear for actions since information in both treatments reduces challenging to a similar extent: challenging is 66% in S and 69% in s+S. Simulations likewise show that fighting in the information treatments is substantively similar (36% in S and 34% in s+S) but different to the no information treatment in which 50% of subject fight.

While the focus of our paper has been on fighting in prisons, since prisoners frequently face the interaction that we test and do so under extreme payoffs, and wall sitting measures toughness and realistically models fighting, our findings may apply to a broader context. They may apply directly to other domains in which fights can easily erupt such as schools and the military, and indirectly to those contexts in which conflicts occur: employees jockeying for a promotion, academics competing for influence, and politicians debating for votes. Further work should be conducted to test the generalizability of our findings, whether they hold with different samples including female and non-student subjects, and whether modifications in the experimental setup substantively affect the results.

## Supporting information

S1 Appendix Model outputs and additional analysis. (DOCX)

S2 Appendix Model analysis and predictions. (DOCX)

S3 Appendix Experimental instructions. (DOCX)
