# Supplementary material for: Does information about toughness decrease fighting? Experimental evidence
Source: PLoS One. 2020 Feb 7;15(2):e0228285. doi: 10.1371/journal.pone.0228285 (PMC7006906; doi:10.1371/journal.pone.0228285)
Supplement: S1 Appendix — (DOCX) [file pone.0228285.s001.docx]

S1 Appendix: Model outputs and additional analysis

Table S1. Predictors of veiled wall sit times.

|  | (1) |
| --- | --- |
|  | Veiled wall sit time (s) |
| Pain resistance | 16.555^**^ |
|  | (5.151) |
|  |  |
| Fitness | 12.897^*^ |
|  | (6.031) |
|  |  |
| 2D:4D | -221.192^*^ |
|  | (104.204) |
|  |  |
| Frequency of exercise | 2.546 |
|  | (4.200) |
|  |  |
| BPAQ | -0.278 |
|  | (0.322) |
|  |  |
| Age | -0.360 |
|  | (1.265) |
|  |  |
| BMI | -2.857^†^ |
|  | (1.555) |
|  |  |
| Constant | 321.479^**^ |
|  | (114.384) |
| Observations | 192 |

Standard errors in parentheses

^†^ *p* < 0.10, ^*^ *p* < 0.05, ^**^ *p* < 0.01, ^***^ *p* < 0.001

To obtain data on 2D:4D, we asked participants to place their hands flat on a table so that we could photograph their palms for later measurement. A number of subjects (46/192), did not place their hands directly flat, which increases measurement error and potentially biases the relationship between 2D:4D and wall sitting. Nevertheless, when we restrict our analysis to the 146 subjects who strictly complied with the hand placement, we find that the relationship between 2D:4D remains significantly negatively linked to wall sitting (b=-265.04, s.e.=123.61, p=0.034).

Table S2. Veterans challenging by their own veiled and unveiled wall sit grades.

|  | (1) | (2) |
| --- | --- | --- |
|  | Veterans challenging | Veterans challenging |
| Veiled grade | 2.882^***^ |  |
|  | (0.459) |  |
|  |  |  |
| Unveiled grade |  | 3.467^***^ |
|  |  | (0.761) |
|  |  |  |
| Constant | 0.304^***^ | 0.389^**^ |
|  | (0.091) | (0.118) |
| Observations | 617 | 617 |

Exponentiated coefficients; Standard errors in parentheses

Standard errors clustered according to 194 subjects

^*^ *p* < 0.05, ^**^ *p* < 0.01, ^***^ *p* < 0.001

Table S3. Rookies resisting by their veiled and unveiled wall sit grades.

|  | (1) | (2) |
| --- | --- | --- |
|  | Rookies resisting | Rookies resisting |
| Veiled grade | 3.106^***^ |  |
|  | (0.635) |  |
|  |  |  |
| Unveiled grade |  | 4.485^***^ |
|  |  | (1.279) |
|  |  |  |
| Constant | 0.129^***^ | 0.139^***^ |
|  | (0.056) | (0.058) |
| Observations | 194 | 194 |

Exponentiated coefficients; Standard errors in parentheses

^*^ *p* < 0.05, ^**^ *p* < 0.01, ^***^ *p* < 0.001

Table S4. Veterans challenging by the sign and signal they observe.

|  | (1) |
| --- | --- |
|  | Veterans ignoring |
| Sign observed | 1.579^***^ |
|  | (0.124) |
|  |  |
| Signal observed | 2.398^***^ |
|  | (0.310) |
|  |  |
| Constant | 0.028^***^ |
|  | (0.012) |
| Observations | 390 |

Exponentiated coefficients; Standard errors in parentheses

Standard errors clustered according to 65 subjects

^*^ *p* < 0.05, ^**^ *p* < 0.01, ^***^ *p* < 0.001

Table S5. Actions by treatment.

|  | (1) | (2) |
| --- | --- | --- |
|  | Challenge | Resist |
| S | 0.523^*^ | 0.607 |
|  | (0.170) | (0.223) |
|  |  |  |
| s+S | 0.593 | 0.694 |
|  | (0.196) | (0.235) |
|  |  |  |
| Constant | 3.706^***^ | 1.581^*^ |
|  | (1.015) | (0.363) |
| Observations | 617 | 194 |

Exponentiated coefficients; Standard errors in parentheses

Standard errors clustered according to 194 subjects in the Challenge model

^*^ *p* < 0.05, ^**^ *p* < 0.01, ^***^ *p* < 0.001

Table S6. Unveiled wall sit times by treatment according to veiled grade obtained.

|  | (1) | (2) | (3) |
| --- | --- | --- | --- |
|  | Unveiled wall sit (s) in NI | Unveiled wall sit (s) in S | Unveiled wall sit (s) in s+S |
| Veiled grade: A | 67.187^***^ | 45.562^*^ | 66.500^***^ |
|  | (15.868) | (18.907) | (16.941) |
|  |  |  |  |
| Veiled grade: B | 12.846 | 23.346 | 37.333^*^ |
|  | (16.160) | (18.627) | (17.714) |
|  |  |  |  |
| Constant | 62.385^***^ | 67.125^***^ | 47.667^***^ |
|  | (11.427) | (13.369) | (12.372) |
| Observations | 80 | 49 | 65 |

Standard errors in parentheses

^*^ *p* < 0.05, ^**^ *p* < 0.01, ^***^ *p* < 0.001
