# Supplementary material for: Does information about toughness decrease fighting? Experimental evidence
Source: PLoS One. 2020 Feb 7;15(2):e0228285. doi: 10.1371/journal.pone.0228285 (PMC7006906; doi:10.1371/journal.pone.0228285)
Supplement: S2 Appendix — (DOCX) [file pone.0228285.s002.docx]

S2 Appendix: Model analysis and predictions

**S2.1 Game 1: No Information**

Consider the following Bayesian game (*G1*), in which there are two players: a Veteran and a Rookie (Fig S1).

Fig S1. Game 1. Blue lines show equilibrium strategies. Expected payoffs displayed.

Nature moves first and chooses a high, medium, or low type Veteran {V_A_, V_B_, V_C_} each with 1/3^rd^ probability and a high, medium, or low type Rookie {R_A_, R_B_, R_C_} also each with 1/3^rd^ probability. Both V and R know the probability of Nature choosing each type.

The Veteran moves second and decides between challenge (*c*) and ignore (*i*). If the Veteran decides to ignore, the interaction ends, while, if the Veteran decides to challenge, the Rookie makes a decision to yield (*y*) or resist (*r*).

The outcome to (*i*, *y*) and (*i*, *r*) is 3, 10. The outcome to (*c*, *y*) is 10, 3 and the outcome to (*c*, *r*) is a fight. The winner of the fight gets 6 and the loser gets 0.

Who wins and who loses in a fight depends on the type of the Veteran and the Rookie (Table S7):

- V_A_ always wins against R_B_ and R_C_, and has a 0.5 probability of winning against a R_A_.
- V_B_ always wins against R_C_, has a 0.5 probability of winning against R_B_, and always loses to R_A_.
- V_C_ wins against R_C_ with a 0.5 probability and always loses against R_B_ and R_A_.

Table S7. Probability of V winning a fight against R depending on type.

|  | R_A_ | R_B_ | R_C_ |
| --- | --- | --- | --- |
| V_A_ | 0.5 | 1 | 1 |
| V_B_ | 0 | 0.5 | 1 |
| V_C_ | 0 | 0 | 0.5 |

Both the Veteran and the Rookie know their own type but not the type of their opponent. A strategy profile therefore consists of six actions, one for each type of Veteran and Rookie.

To identify the pure-strategy perfect Bayesian equilibria (PBE) in *G1* we (i) calculate expected utilities to all pure strategy profiles (see Table S8), (ii) find pure strategy Nash equilibria (NE) based on expected utilities, and (iii) to check whether the identified NE are also PBE, we specify passive conjectures as out-of-equilibrium beliefs for Rookies (i.e. 1/3rd probability that each type of Veteran challenges).

Table S8. Expected utilities for *G1*.

| V_A_, V_B_, V_C_ | R_A_, R_B_, R_C_ | | | | | | | |
| --- | --- | --- | --- | --- | --- | --- | --- | --- |
|  | *y, y, y* | *y, y, r* | *y, r, y* | *r, y, y* | *y, r, r* | *r, y, r* | *r, r, y* | *r, r, r* |
| *c, c, c* | **10**, **10**, **10**  3, **3**, **3** | **8.7**, **8.7**, **7.7**  3, **3**, 1 | **8.7**, **7.7**, **6.6**  3, **3**, **3** | **7.7, 6.6, 6.6**  **5, 3, 3** | **7.3**, **6.3**, **4.3**  3, **3**, 1 | **6.3**, **5.3**, **4.3**  **5**, **3**, 1 | **6.3, 4.3, 3.3**  **5, 3, 3** | **5**, **3**, 1  **5**, **3**, 1 |
| *c, c, i* | **10**, **10**, 3  5.3, **5.3**, **5.3** | **8.7**, **8.7**, 3  5.3, **5.3**, 3.3 | **8.7**, **7.7**, 3  5.3, 4.3, **5.3** | **7.7**, **6.6**, 3  **6.3**, **5.3**, **5.3** | **7.3**, **6.3**, 3  5.3, 4.3, 3.3 | **6.3**, **5.3**, 3  **6.3**, **5.3**, 3.3 | **6.3**, 4.3, 3  **6.3**, 4.3, **5.3** | **5**, **3**, **3**  **6.3**, 4.3, 3.3 |
| *c, i, c* | **10**, 3, **10**  5.3, **5.3**, **5.3** | **8.7**, 3, **7.7**  5.3, **5.3**, 4.3 | **8.7**, 3, **6.6**  5.3, **5.3**, **5.3** | **7.7**, 3, **6.6**  **6.3**, **5.3**, **5.3** | **7.3**, 3, **4.3**  5.3, **5.3**, 4.3 | **6.3**, 3, **4.3**  **6.3**, **5.3**, 4.3 | **6.3**, 3, **3.3**  **6.3**, **5.3**, **5.3** | **5**, **3**, 1  **6.3**, **5.3**, 4.3 |
| *i, c, c* | 3, **10**, **10**  5.3, 5.3, **5.3** | 3, **8.7**, **7.7**  5.3, 5.3, 4.3 | 3, **7.7**, **6.6**  5.3, **6.3**, **5.3** | 3, **6.6**, **6.6**  **7.3**, 5.3, **5.3** | 3, **6.3**, **4.3**  5.3, **6.3**, 4.3 | 3, **5.3**, **4.3**  **7.3**, 5.3, 4.3 | 3, **4.3**, **3.3**  **7.3**, **6.3**, **5.3** | 3, **3**, 1  **7.3**, **6.3**, 4.3 |
| *c, i, i* | **10**, 3, 3  **7.7**, **7.7**, **7.7** | **8.7**, 3, 3  **7.7**, **7.7**, 6.7 | **8.7**, 3, 3  **7.7**, 6.7, **7.7** | **7.7**, 3, 3  **7.7**, **7.7**, **7.7** | **7.3**, 3, 3  **7.7**, 6.7, 6.7 | **6.3**, 3, 3  **7.7**, **7.7**, 6.7 | **6.3**, 3, 3  **7.7**, 6.7, **7.7** | **5**, **3**, **3**  **7.7**, 6.7, 6.7 |
| *i, c, i* | 3, **10**, 3  7.7, **7.7**, **7.7** | 3, **8.7**, 3  7.7, **7.7**, 6.7 | 3, **7.7**, 3  7.7, **7.7**, **7.7** | 3, **6.6**, 3  **8.7**, **7.7**, **7.7** | 3, **6.3**, 3  7.7, **7.7**, 6.7 | 3, **5.3**, 3  **8.7**, **7.7**, 6.7 | 3, **4.3**, 3  **8.7**, **7.7**, **7.7** | 3, **3**, **3**  **8.7**, **7.7**, 6.7 |
| *i, i, c* | 3, 3, **10**  7.7, 7.7, **7.7** | 3, 3, **7.7**  7.7, 7.7, **7.7** | 3, 3, **6.6**  7.7, **8.7**, **7.7** | 3, 3, **6.6**  **8.7**, 7.7, **7.7** | 3, 3, **4.3**  7.7, **8.7**, **7.7** | 3, 3, **4.3**  **8.7**, 7.7, **7.7** | 3, 3, **3.3**  **8.7**, **8.7**, **7.7** | 3, **3**, 1  **8.7**, **8.7**, **7.7** |
| *i, i, i* | 3, 3, 3  **10**, **10**, **10** | 3, 3, 3  **10**, **10**, **10** | 3, 3, 3  **10**, **10**, **10** | 3, 3, 3  **10**, **10**, **10** | 3, 3, 3  **10**, **10**, **10** | 3, 3, 3  **10**, **10**, **10** | 3, 3, 3  **10**, **10**, **10** | 3, **3**, **3**  **10**, **10**, **10** |

*Note:* The outcomes associate with best response actions are indicated in bold. The outcomes associated with pure strategy equilibria, strategy profiles in which all players are playing a best response, are underlined.

We find two pure strategy NE in *G1*: {(*c*, *c*, *c*); (*r*, *y*, *y*)} and {(*c*, *c*, *c*); (*r*, *r*, *y*)} (see Table S8). Both NE are also PBE with passive conjectures for out-of-equilibrium beliefs.

Consider now the outcomes that occur in each equilibrium. Start with {(*c*, *c*, *c*); (*r*, *y*, *y*)} (Table S9)— interestingly, we see here that weaker prisoners (V_C_) exploit tougher rookies (R_B_); a result that also occurs in the equilibria in *G2*. This implies that fights occur between all V-types and R_A_ and that 33% of interactions end in fighting. In 1/6^th^ of these fights V win and in 5/6^th^ R win. It also implies that 67% of interactions result in exploitation (all V-types against R_B_ and R_C_) and that conditional on not fighting, there is 100% exploitation. 0% of interactions end in status quo: either there is fighting or there is exploitation.

Table S9. Outcomes in the pure strategy equilibrium {(c, c, c); (r, y, y)}.

| Veterans | Rookies |  |  |
| --- | --- | --- | --- |
|  | R_A_ | R_B_ | R_C_ |
| V_A_ | Fight (V or R win) | Exploitation | Exploitation |
| V_B_ | Fight (R win) | Exploitation | Exploitation |
| V_C_ | Fight (R win) | Exploitation | Exploitation |

The other equilibrium {(*c*, *c*, *c*); (*r*, *r*, *y*)} implies that fights occur between all V-types and R_A_ and R_B_, and thus fighting is the outcome in 67% of interactions (Table S10). In 2/6^th^ of these fights V win and in 4/6^th^ R win. Exploitation occurs in the other 33% of interactions (all V-types against R_C_), or, conditional on not fighting, there is 100% exploitation. Likewise, 0% of interactions end in status quo: either there is fighting or there is exploitation.

Table S10. Outcomes in the pure strategy equilibrium {(c, c, c); (r, r, y)}.

| Veterans | Rookies |  |  |
| --- | --- | --- | --- |
|  | R_A_ | R_B_ | R_C_ |
| V_A_ | Fight (V or R win) | Fight (V win) | Exploitation |
| V_B_ | Fight (R win) | Fight (V or R win) | Exploitation |
| V_C_ | Fight (R win) | Fight (R win) | Exploitation |

**S2.2 Game 2: V Know R Toughness**

Now consider *G2*. This game is identical to *G1* except in one respect: Veterans now observe the signs and signals of the Rookies which allows them to infer whether Rookies are high, medium, or low type—Rookies still do not know the type of Veterans (Fig S2). Rookies know that Veterans know Rookie’s type.

A strategy profile therefore now consists of 12 actions. Each type of Veteran takes an action against each different type of Rookie (thus 3*3 actions) and each Rookie type makes a single action against all Veterans (3 actions).

Before searching for equilibria, we simplify the strategy space using iterative deletion of strictly dominated strategies:

1. For V_A_ against R_C_ and R_B_, *c* strictly dominates *i.* If R_C_ or R_B_ choose to *y*, V_A_ gains 10 from *c* and 3 from *i*. If R_C_ or R_B_ decide to *R* then V_A_ gains 6 by *c* (due to subsequently winning in a fight) and 3 from *i*.
2. *c* for V_B_ against R_C_ strictly dominates *i*. If R_C_ decides to *y*, V_B_ gains 10 by *c* and 3 by *i*. If R_C_ chooses to *r*, V_B_ gains 6 from *c* and 3 from *i*.
3. *y* for R_C_ strictly dominates *r* in all strategy profiles in which there is some proportion > 0 of V_A_ and V_B_ who *c* R_C_. When the proportion of V_A_ and/or V_B_ who *c* R_C_ is > 0, then R_C_ receives 3 from *y* and < 3 from *r*, since, R_C_ always loses in a fight with V_A_ and V_B_. By proposition 1 and/or 2 this must be the case in equilibrium. (Only when the proportion of V_A_ and V_B_ *c* R_C_ is = 0, then R_C_ is indifferent between R and *y* irrespective of the action of V_C_. However, this cannot be the case in equilibrium since V_A_ and V_B_ strictly prefer to *c* R_C_ than to *i* R_C_.)
4. Since proposition 3, V_C_ strictly prefers to *c* R_C_ than to *i* R_C_ (because 10>3).

Following the elimination of the above strategies, we use the same approach as in *G1*. We then assume passive conjectures as out-of-equilibrium beliefs for Rookies (i.e. 1/3rd probability that each type of Veteran challenges).

Fig S2. Game 2. Blue lines show equilibrium strategies. Expected payoffs displayed.

Table S11. Expected utilities for G2.

| V_A_\|R_A_, V_A_\|R_B_, V_A_\|R_C_, V_B_\|R_A_, V_B_\|R_B_, V_B_\|R_C_, V_C_\|R_A_, V_C_\|R_B_, V_C_\|R_C_ | | | R_A_, R_B_, R_C_ | | | |
| --- | --- | --- | --- | --- | --- | --- |
|  |  |  | *y, y, y* | *y, r, y* | *r, y, y* | *r, r, y* |
| *c, c, c,* | *c, c, c,* | *c, c, c* | **10**, **10**, **10**, **10**, **10**, **10**, **10**, **10**, **10**  3, **3**, **3** | **10**, **6**, **10**, **10**, **3**, **10**, **10**, 0, **10**  3, **3**, 3 | **3**, **10**, **10**, 0, **10**, **10**, 0, **10**, **10**  **5**, **3**, **3** | **3**, **6**, **10**, 0, **3**, **10**, 0, 0, **10**  **5**, **3**, **3** |
| *c, c, c,* | *c, c, c,* | *c, i, c* | **10**, **10**, **10**, **10**, **10**, **10**, **10**, 3, **10**  3, **5.3**, **3** | **10**, **6**, **10**, **10**, **3**, **10**, **10**, **3**, **10**  3, 4.3, **3** | **3**, **10**, **10**, 0, **10**, **10**, 0, 3, **10**  **5**, **5.3**, **3** | **3**, **6**, **10**, 0, **3**, **10**, 0, **3**, **10**  **5**, 4.3, **3** |
| *c, c, c,* | *c, c, c,* | *i, c, c* | **10**, **10**, **10**, **10**, **10**, **10**, 3, **10**, **10**  5.3, **3**, **3** | **10**, **6**, **10**, **10**, **3**, **10**, 3, 0, **10**  5.3, **3**, **3** | **3**, **10**, **10**, 0, **10**, **10**, **3**, **10**, **10**  **6.3**, **3**, **3** | **3**, **6**, **10**, 0, **3**, **10**, **3**, 0, **10**  **6.3**, **3**, **3** |
| *c, c, c,* | *c, i, c,* | *c, c, c* | **10**, **10**, **10**, **10**, 3, **10**, **10**, **10**, **10**  3, **5.3**, **3** | **10**, **6**, **10**, **10**, **3**, **10**, **10**, 0, **10**  3, **5.3**, **3** | **3**, **10**, **10**, 0, 3, **10**, 0, **10**, **10**  **5**, **5.3**, **3** | **3**, **6**, **10**, 0, **3**, **10**, 0, 0, **10**  **5**, **5.3**, **3** |
| *c, c, c,* | *i, c, c,* | *c, c, c* | **10**, **10**, **10**, 3, **10**, **10**, **10**, **10**, **10**  5.3, **3**, **3** | **10**, **6**, **10**, 3, **3**, **10**, **10**, 0, **10**  5.3, **3**, **3** | **3**, **10**, **10**, **3**, **10**, **10**, 0, **10**, **10**  **6.3**, **3**, **3** | **3**, **6**, **10**, **3**, **3**, **10**, 0, 0, **10**  **6.3**, **3**, **3** |
| *i, c, c,* | *c, c, c,* | *c, c, c* | 3, **10**, **10**, **10**, **10**, **10**, **10**, **10**, **10**  5.3, **3**, **3** | 3, **6**, **10**, **10**, **3**, **10**, **10**, 0, **10**  5.3, **3**, **3** | **3**, **10**, **10**, 0, **10**, **10**, 0, **10**, **10**  **7.3**, **3**, **3** | **3**, **6**, **10**, 0, **3**, **10**, 0, 0, **10**  **7.3**, **3**, **3** |
| *c, c, c,* | *c, c, c,* | *i, i, c* | **10**, **10**, **10**, **10**, **10**, **10**, 3, 3, **10**  5.3, **5.3**, **3** | **10**, **6**, **10**, **10**, **3**, **10**, 3, **3**, **10**  5.3, 4.3, **3** | **3**, **10**, **10**, 0, **10**, **10**, **3**, 3, **10**  **6.3**, **5.3**, **3** | **3**, **6**, **10**, 0, **3**, **10**, **3**, **3**, **10**  **6.3**, 4.3, **3** |
| *c, c, c,* | *c, i, c,* | *i, c, c* | **10**, **10**, **10**, **10**, 3, **10**, 3, **10**, **10**  5.3, **5.3**, **3** | **10**, **6**, **10**, **10**, **3**, **10**, 3, 0, **10**  5.3, **5.3**, **3** | **3**, **10**, **10**, 0, 3, **10**, **3**, **10**, **10**  **6.3**, **5.3**, **3** | **3**, **6**, **10**, 0, **3**, **10**, **3**, 0, **10**  **6.3**, **5.3**, **3** |
| *c, c, c,* | *i, i, c,* | *c, c, c* | **10**, **10**, **10**, 3, 3, **10**, **10**, **10**, **10**  5.3, **5.3**, **3** | **10**, **6**, **10**, 3, **3**, **10**, **10**, 0, **10**  5.3, **5.3**, **3** | **3**, **10**, **10**, **3**, 3, **10**, 0, **10**, **10**  **6.3**, **5.3**, **3** | **3**, **6**, **10**, **3**, **3**, **10**, 0, 0, **10**  **6.3**, **5.3**, **3** |
| *i, c, c,* | *i, c, c,* | *c, c, c* | 3, **10**, **10**, 3, **10**, **10**, **10**, **10**, **10**  7.7, **3**, **3** | 3, **6**, **10**, 3, **3**, **10**, **10**, 0, **10**  7.7, **3**, **3** | **3**, **10**, **10**, **3**, **10**, **10**, 0, **10**, **10**  **8.7**, **3**, **3** | **3**, **6**, **10**, **3**, **3**, **10**, 0, 0, **10**  **8.7**, **3**, **3** |
| *c, c, c,* | *c, i, c,* | *c, i, c* | **10**, **10**, **10**, **10**, 3, **10**, **10**, 3, **10**  3, **7.7**, **3** | **10**, **6**, **10**, **10**, **3**, **10**, **10**, **3**, **10**  3, 6.7, **3** | **3**, **10**, **10**, 0, 3, **10**, 0, 3, **10**  **5**, **7.7**, **3** | **3**, **6**, **10**, 0, **3**, **10**, 0, **3**, **10**  **5**, 6.7, **3** |
| *c, c, c,* | *i, c, c,* | *i, c, c* | **10**, **10**, **10**, 3, **10**, **10**, 3, **10**, **10**  **7.7**, **3**, **3** | **10**, **6**, **10**, 3, **3**, **10**, 3, 0, **10**  **7.7**, **3**, **3** | **3**, **10**, **10**, **3**, **10**, **10**, **3**, **10**, **10**  **7.7**, **3**, **3** | **3**, **6**, **10**, **3**, **3**, **10**, **3**, 0, **10**  **7.7**, **3**, **3** |
| *i, c, c,* | *c, i, c,* | *c, c, c* | 3, **10**, **10**, **10**, 3, **10**, **10**, **10**, **10**  5.3, **5.3**, **3** | 3, **6**, **10**, **10**, **3**, **10**, **10**, 0, **10**  5.3, **5.3**, **3** | **3**, **10**, **10**, 0, 3, **10**, 0, **10**, **10**  **7.3**, **5.3**, **3** | **3**, **6**, **10**, 0, **3**, **10**, 0, 0, **10**  **7.3**, **5.3**, **3** |
| *c, c, c,* | *i, c, c,* | *c, i, c* | **10**, **10**, **10**, 3, **10**, **10**, **10**, 3, **10**  5.3, **5.3**, **3** | **10**, **6**, **10**, 3, **3**, **10**, **10**, **3**, **10**  5.3, 4.3, **3** | **3**, **10**, **10**, **3**, **10**, **10**, 0, 3, **10**  **6.3**, **5.3**, **3** | **3**, **6**, **10**, **3**, **3**, **10**, 0, **3**, **10**  **6.3**, 4.3, **3** |
| *i, c, c,* | *c, c, c,* | *c, i, c* | 3, **10**, **10**, **10**, **10**, **10**, **10**, 3, **10**  5.3, **5.3**, **3** | 3, **6**, **10**, **10**, **3**, **10**, **10**, **3**, **10**  5.3, 4.3, **3** | **3**, **10**, **10**, 0, **10**, **10**, 0, 3, **10**  **7.3**, **5.3**, **3** | **3**, **6**, **10**, 0, **3**, **10**, 0, **3**, **10**  **7.3**, 4.3, **3** |
| *i, c, c,* | *c, c, c,* | *i, c, c* | 3, **10**, **10**, **10**, **10**, **10**, 3, **10**, **10**  7.7, **3**, **3** | 3, **6**, **10**, **10**, **3**, **10**, 3, 0, **10**  7.7, **3**, **3** | **3**, **10**, **10**, 0, **10**, **10**, **3**, **10**, **10**  **8.7**, **3**, **3** | **3**, **6**, **10**, 0, **3**, **10**, **3**, 0, **10**  **8.7**, **3**, **3** |
| *i, c, c,* | *i, i, c,* | *c, c, c* | 3, **10**, **10**, 3, 3, **10**, **10**, **10**, **10**  7.7, **5.3**, **3** | 3, **6**, **10**, 3, **3**, **10**, **10**, 0, **10**  7.7, **5.3**, **3** | **3**, **10**, **10**, **3**, 3, **10**, 0, **10**, **10**  **8.7**, **5.3**, **3** | **3**, **6**, **10**, **3**, **3**, **10**, 0, 0, **10**  **8.7**, **5.3**, **3** |
| *i, c, c,* | *i, c, c,* | *c, i, c* | 3, **10**, **10**, 3, **10**, **10**, **10**, 3, **10**  7.7, **5.3**, **3** | 3, **6**, **10**, 3, **3**, **10**, **10**, **3**, **10**  7.7, 4.3, **3** | **3**, **10**, **10**, **3**, **10**, **10**, 0, 3, **10**  **8.7**, **5.3**, **3** | **3**, **6**, **10**, **3**, **3**, **10**, 0, **3**, **10**  **8.7**, 4.3, **3** |
| *i, c, c,* | *c, c, c,* | *i, i, c* | 3, **10**, **10**, **10**, **10**, **10**, 3, 3, **10**  7.7, **5.3**, **3** | 3, **6**, **10**, **10**, **3**, **10**, 3, **3**, **10**  7.7, 4.3, **3** | **3**, **10**, **10**, 0, **10**, **10**, **3**, 3, **10**  **8.7**, **5.3**, **3** | **3**, **6**, **10**, 0, **3**, **10**, **3**, **3**, **10**  **8.7**, 4.3, **3** |
| *c, c, c,* | *c, i, c,* | *i, i, c* | **10**, **10**, **10**, **10**, 3, **10**, 3, 3, **10**  5.3, **7.7**, **3** | **10**, **6**, **10**, **10**, **3**, **10**, 3, **3**, **10**  5.3, 6.7, **3** | **3**, **10**, **10**, 0, 3, **10**, **3**, 3, **10**  **6.3**, **7.7**, **3** | **3**, **6**, **10**, 0, **3**, **10**, **3**, **3**, **10**  **6.3**, 6.7, **3** |
| *i, c, c,* | *i, c, c,* | *i, c, c* | 3, **10**, **10**, 3, **10**, **10**, 3, **10**, **10**  **10**, **3**, **3** | 3, **6**, **10**, 3, **3**, **10**, 3, 0, **10**  **10**, **3**, **3** | **3**, **10**, **10**, **3**, **10**, **10**, **3**, **10**, **10**  **10**, **3**, **3** | **3**, **6**, **10**, **3**, **3**, **10**, **3**, 0, **10**  **10**, **3**, **3** |
| *i, c, c,* | *c, i, c,* | *c, i, c* | 3, **10**, **10**, **10**, 3, **10**, **10**, 3, **10**  5.3, **7.7**, **3** | 3, **6**, **10**, **10**, **3**, **10**, **10**, **3**, **10**  5.3, 6.7, **3** | **3**, **10**, **10**, 0, 3, **10**, 0, 3, **10**  **7.3**, **7.7**, **3** | **3**, **6**, **10**, 0, **3**, **10**, 0, **3**, **10**  **7.3**, 6.7, **3** |
| *c, c, c,* | *i, c, c,* | *i, i, c* | **10**, **10**, **10**, 3, **10**, **10**, 3, 3, **10**  **7.7**, **5.3**, **3** | **10**, **6**, **10**, 3, **3**, **10**, 3, **3**, **10**  **7.7**, 4.3, **3** | **3**, **10**, **10**, **3**, **10**, **10**, **3**, 3, **10**  **7.7**, **5.3**, **3** | **3**, **6**, **10**, **3**, **3**, **10**, **3**, **3**, **10**  **7.7**, 4.3, **3** |
| *i, c, c,* | *c, i, c,* | *i, c, c* | 3, **10**, **10**, **10**, 3, **10**, 3, **10**, **10**  7.7, **5.3**, **3** | 3, **6**, **10**, **10**, **3**, **10**, 3, 0, **10**  7.7, **5.3**, **3** | **3**, **10**, **10**, 0, 3, **10**, **3**, **10**, **10**  **8.7**, **5.3**, **3** | **3**, **6**, **10**, 0, **3**, **10**, **3**, 0, **10**  **8.7**, **5.3**, **3** |
| *c, c, c,* | *i, i, c,* | *i, c, c* | **10**, **10**, **10**, 3, 3, **10**, 3, **10**, **10**  **7.7**, **5.3**, **3** | **10**, **6**, **10**, 3, **3**, **10**, 3, 0, **10**  **7.7**, **5.3**, **3** | **3**, **10**, **10**, **3**, 3, **10**, **3**, **10**, **10**  **7.7**, **5.3**, **3** | **3**, **6**, **10**, **3**, **3**, **10**, **3**, 0, **10**  **7.7**, **5.3**, **3** |
| *c, c, c,* | *i, i, c,* | *c, i, c* | **10**, **10**, **10**, 3, 3, **10**, **10**, 3, **10**  5.3, **7.7**, **3** | **10**, **6**, **10**, 3, **3**, **10**, **10**, **3**, **10**  5.3, 6.7, **3** | **3**, **10**, **10**, **3**, 3, 10, 0, 3, **10**  **6.3**, **7.7**, **3** | **3**, **6**, **10**, **3**, **3**, **10**, 0, **3**, **10**  **6.3**, 6.7, **3** |
| *i, c, c,* | *i, i, c,* | *i, c, c* | 3, **10**, **10**, 3, 3, **10**, 3, **10**, **10**  **10**, **5.3**, **3** | 3, **6**, **10**, 3, **3**, **10**, 3, 0, **10**  **10**, **5.3**, **3** | **3**, **10**, **10**, **3**, 3, **10**, **3**, **10**, **10**  **10**, **5.3**, **3** | **3**, **6**, **10**, **3**, **3**, **10**, **3**, 0, **10**  **10**, **5.3**, **3** |
| *i, c, c,* | *i, i, c,* | *c, i, c* | 3, **10**, **10**, 3, 3, **10**, **10**, 3, **10**  7.7, **7.7**, **3** | 3, **6**, **10**, 3, **3**, **10**, **10**, **3**, **10**  7.7, 6.7, **3** | **3**, **10**, **10**, **3**, 3, **10**, 0, 3, **10**  **8.7**, **7.7**, **3** | **3**, **6**, **10**, **3**, **3**, **10**, 0, **3**, **10**  **8.7**, 6.7, **3** |
| *i, c, c,* | *i, c, c,* | *i, i, c* | 3, **10**, **10**, 3, **10**, **10**, 3, 3, **10**  **10**, **5.3**, **3** | 3, **6**, **10**, 3, **3**, **10**, 3, **3**, **10**  **10**, 4.3, **3** | **3**, **10**, **10**, **3**, **10**, **10**, **3**, 3, **10**  **10**, **5.3**, **3** | **3**, **6**, **10**, **3**, **3**, **10**, **3**, **3**, **10**  **10**, 4.3, **3** |
| *i, c, c,* | *c, i, c,* | *i, i, c* | 3, **10**, **10**, **10**, 3, **10**, 3, 3, **10**  7.7, **7.7**, **3** | 3, **6**, **10**, **10**, **3**, **10**, 3, **3**, **10**  7.7, 6.7, **3** | **3**, **10**, **10**, 0, 3, **10**, **3**, 3, **10**  **8.7**, **7.7**, **3** | **3**, **6**, **10**, 0, **3**, **10**, **3**, **3**, **10**  **8.7**, 6.7, **3** |
| *c, c, c,* | *i, i, c,* | *i, i, c* | **10**, **10**, **10**, 3, 3, **10**, 3, 3, **10**  **7.7**, **7.7**, **3** | **10**, **6**, **10**, 3, **3**, **10**, 3, **3**, **10**  **7.7**, 6.7, **3** | **3**, **10**, **10**, **3**, 3, **10**, **3**, 3, **10**  **7.7**, **7.7**, **3** | **3**, **6**, **10**, **3**, **3**, **10**, **3**, **3**, **10**  **7.7**, 6.7, **3** |
| *i, c, c,* | *i, i, c,* | *i, i, c* | 3, **10**, **10**, 3, 3, **10**, 3, 3, **10**  **10**, **7.7**, **3** | 3, **6**, **10**, 3, **3**, **10**, 3, **3**, **10**  **10**, 6.7, **3** | **3**, **10**, **10**, **3**, 3, **10**, **3**, 3, **10**  **10**, **7.7**, **3** | **3**, **6**, **10**, **3**, **3**, **10**, **3**, **3**, **10**  **10**, 6.7, **3** |

*Note:* The outcomes associate with best response actions are indicated in bold. The outcomes associated with pure strategy equilibria, strategy profiles in which all players are playing a best response, are underlined.

There are only two pure strategy NE (Table S11). These are {(*c, c, c, i, c, c, i, c, c*); (*r, y, y*)} and {(*i, c, c, i, c, c, i, c, c*); (*r, y, y*)}. Both NE are also PBE when we posit passive conjectures as out-of-equilibrium beliefs.

The first NE, {(*c, c, c, i, c, c, i, c, c*); (*r, y, y*)}, implies that 11% of interactions end in fighting (Table S12). Only R_A_ decide to *r*, while the other R-types choose to *y*. Among Veterans, only V_A_ *c* R_A_. This means that there is fighting only when a high-type V and a high type-type R meet which occurs in 1/9^th^ of the interactions (half of these fights R win and half V win). In this same NE, 67% of interactions end in exploitation, and conditional on not fighting 75% of interactions end in exploitation. 22% of interactions end in status quo, and conditional on challenging, 86% end in exploitation.

Table S12. Outcomes in the pure strategy equilibrium {(c, c, c, i, c, c, i, c, c); (r, y, y)}.

| Veterans | Rookies |  |  |
| --- | --- | --- | --- |
|  | R_A_ | R_B_ | R_C_ |
| V_A_ | Fight (V or R win) | Exploitation | Exploitation |
| V_B_ | Status quo | Exploitation | Exploitation |
| V_C_ | Status quo | Exploitation | Exploitation |

In the second NE {(*i, c, c, i, c, c, i, c, c*); (*r, y, y*)} there is no fighting (Table S13). Even V_A_ choose to *i* R_A_. For exploitation 67% of interactions end this way (conditional and unconditional on fighting is the same since there is no fighting). (One may wonder why R_B_ do not *r*? V who decide to *c* R_B_ are either only V_A_ or V_A_ and some proportion of V_B_. In this case if R_B_ chooses to *r* he gains 0 from fights with V_A_ and 3 from fights with V_B_, while he gains 3 from encounters with V_A_ and 3 from encounters with V_B_ if he decides to *y*.) 33% of interactions end in status quo and conditional on challenging, 100% are in exploitation.

Table S13. Outcomes in the pure strategy equilibrium {(i, c, c, i, c, c, i, c, c); (r, y, y)}.

| Veterans | Rookies |  |  |
| --- | --- | --- | --- |
|  | R_A_ | R_B_ | R_C_ |
| V_A_ | Status quo | Exploitation | Exploitation |
| V_B_ | Status quo | Exploitation | Exploitation |
| V_C_ | Status quo | Exploitation | Exploitation |

Based on this analysis, we derive our hypotheses. We find that when Veterans know the toughness of Rookies:

1. Fighting decreases.
2. Fighting decreases among Veterans and Rookies of different toughness but, not necessarily among Veterans and Rookies of the same (high) toughness.
3. Exploitation decreases.
