# Supplementary material for: Does information about toughness decrease fighting? Experimental evidence
Source: PLoS One. 2020 Feb 7;15(2):e0228285. doi: 10.1371/journal.pone.0228285 (PMC7006906; doi:10.1371/journal.pone.0228285)
Supplement: S3 Appendix — (DOCX) [file pone.0228285.s003.docx]

S3 Appendix: Experimental instructions

The instructions below are the English translations of the Italian-language ones used in the experiment. We include the instructions for the most complex, sign and signal, treatment.

The following instructions were used in the second session for any sit-offs. As before, these are the English translations of the Italian originals.
